# An integrated analysis of prognostic and immune infiltrates for hub genes as potential survival indicators in patients with lung adenocarcinoma

**DOI:** 10.1186/s12957-022-02543-z

**Published:** 2022-03-30

**Authors:** Zhiyun Xu, Shi Wang, Zhijian Ren, Xiang Gao, Lin Xu, Shuai Zhang, Binhui Ren

**Affiliations:** 1 Department of Thoracic Surgery, The Affiliated Cancer Hospital of Nanjing Medical University & Jiangsu Cancer Hospital & Jiangsu Institute of Cancer Research, Nanjing, 210000 China; 2 Department of Cardiothoracic Surgery, The Affiliated Huaian No. 1 People’s Hospital of Nanjing Medical University, Huaian, 223300 China; 3 Department of Cardiothoracic Surgery, Jinling Hospital, Medical School of Nanjing University, Nanjing University, Nanjing, 210000 China

**Keywords:** Lung adenocarcinoma, Tumor microenvironment, Prognosis, Data mining

## Abstract

**Objective:**

Lung adenocarcinoma (LUAD) is one of the major subtypes of lung cancer that is associated with poor prognosis. The aim of this study was to identify useful biomarkers to enhance the treatment and diagnosis of LUAD.

**Methods:**

GEO2R was used to identify common up-regulated differentially expressed genes (DEGs) in the GSE32863, GSE40791, and GSE75037 datasets. The DEGs were submitted to Metascape for gene ontology and pathway enrichment analysis as well as construction of the protein-protein interaction (PPI) network, while the molecular complex detection (MCODE) plug-in was employed to filter important subnetworks. The expression levels of the hub genes and their prognostic values were evaluated using the UALCAN, GEPIA2, and Kaplan-Meier plotter databases. The timer algorithm was utilized to determine the correlation between immune cell infiltration and the expression levels of hub genes in LUAD tissues. In addition, the hub gene mutation landscape and the correlation analysis with tumor mutational burden (TMB) score were evaluated using maftools package and ggstatsplot package in R software, respectively.

**Results:**

We identified 156 common up-regulated DEGs, with gene ontology and pathway enrichment analysis indicating that they were mostly enriched in mitotic cell cycle process and cell cycle pathway. DEGs in the subnetwork with the largest number of genes were AURKB, CCNB2, CDC20, CDCA5, CDCA8, CENPF, and KNTC1. The seven hub genes were highly expressed in LUAD tissues and were associated with poor prognosis. These hub genes were negatively correlated with most immune cells. The somatic mutation landscape showed that AURKB, CDC20, CENPF, and KNTC1 had mutations and were positively correlated with TMB scores.

**Conclusions:**

Our findings demonstrate that increased expression of seven hub genes is associated with poor prognosis for LUAD patients. Additionally, the TMB score indicates that the high expression of hub gene increases immune cell infiltration in patients with lung adenocarcinoma which may significantly improve response to immunotherapy.

**Supplementary Information:**

The online version contains supplementary material available at 10.1186/s12957-022-02543-z.

## Introduction

Lung cancer is the leading cause of cancer-related morbidity and mortality worldwide and is mainly divided into non-small cell lung cancer and small cell lung cancer [1]. The proportion of lung adenocarcinoma (LUAD) in non-small cell lung cancer is approximately 55% [2]. Recently, there has been a significant increase in the incidence of LUAD, but the specific pathogenesis of LUAD still remains unclear [3]. Despite the availability of several therapeutic options for patients with LUAD, including surgery, targeted therapy, and immunotherapy, the overall survival rate of patients is still poor [4, 5]. As a result, it is critical to identify effective biomarkers associated with the progression of LUAD that could serve as therapeutic targets.

The recent advancement in chip sequencing technology has generated a lot of microarray data from various tumor samples [6]. Bioinformatics techniques have been extensively utilized in cancer research to identify useful biomarkers using large amounts of microarray datasets [7, 8]. The purpose of this research was to analyze three LUAD microarray datasets from the GEO database, namely GSE32863, GSE40791, and GSE75037. GEO2R algorithms were used to identify differentially expressed genes (DEGs) between tumor and normal tissues. The common up-regulated genes were then analyzed in Metascape for gene ontology and pathway enrichment [9, 10]. Metascape was also used for PPI network construction, and the MCODE online plug-in was utilized to identify the significant subnetworks of the PPI network [9]. The subnetwork with the greatest number of hub genes was chosen for further analysis. We compared the expression levels of key genes between tumor and normal samples using the GEPIA2 and UALCAN datasets [11, 12] and determined their prognostic value for patients with LUAD using the Kaplan-Meier Plotter database [13]. Finally, the Timer database was used to explore the correlation between hub genes and the infiltration of six different types of immune cells in LUAD tissues, including B cells, CD4+ T cells, CD8+ T cells, neutrophils, macrophages, and dendritic cells [14]. Our aim was to identify useful prognostic markers and immune-related therapeutic targets.

## Methods

### Acquisition of data

All GSE datasets analyzed were downloaded from the GEO database—an open access database for storing microarray and high-throughput sequencing data [15]. The GSE32863 dataset contained data for 58 LUAD tissues and 58 adjacent normal lung tissues analyzed using the Illumina HumanWG-6 v3.0 expression beadchip; the GSE40791 dataset contained data for 94 LUAD samples and 100 normal samples analyzed using the Affymetrix Human Genome U133 Plus 2.0 Array, while the GSE75037 dataset contained data for 83 LUAD tissues and 83 adjacent normal tissues analyzed using the Illumina HumanWG-6 v3.0 expression beadchip. In GSE32863 and GSE75037, each pair of tissues came from the same patient, but in GSE40791, each sample was from each patient. Therefore, a total of 335 patients were included in this study.

### Analysis of DEGs

GEO2R was used to identify DEGs in the three GSE datasets with the parameters set as *P* > 0.05 and |logFC| > 2. A volcano map was used to visualize the DEGs, while a Venn diagram was used to identify up-regulated and down-regulated DEGs in the three GSE datasets. In this study, we were interested in the up-regulated DEGs that were common among the three datasets.

### Analysis of gene ontology and pathway enrichment

Gene ontology (GO) has been widely used to analyze specific functions of genes classified into molecular function (MF), biological process (BP), and cellular component (CC) after annotating a given gene list. The purpose of pathway enrichment analysis is to use statistical methods to find significantly enriched pathway analysis in the target gene list. Metascape is an online tool for GO and biological pathway enrichment analysis, which presents results in form of high-quality charts and sententious explanation [9]. We used Metascape for GO analysis of the common up-regulated DEGs with the cutoff of *P* value, min overlap, and min enrichment set as less than 0.01, 3, and 1.5, respectively.

### Construction of the PPI network

We also used Metascape to construct the PPI network—the interaction network of all proteins based on the relevance and similarity of the submitted gene list. We then selected the significant subnetworks from the overall PPI for subsequent analysis using the MCODE plug-in. We investigated the subnetwork with the largest number of genes and explored the characteristics of its constituent hub genes.

### Verification of the expression level of hub genes

GEPIA2 is a database that can analyze RNA sequencing expression data from 9736 tumor samples and 8587 normal samples from the TCGA and GTEx projects [11], while UALCAN is a comprehensive and interactive online database that validates the expression level of candidate genes in different tumors in the TCGA database [12]. The GEPIA2 database was used to verify the differential expression of hub genes between the tumor tissues and adjacent tissues of LUAD patients, while the UALCAN database was used to analyze the expression level of hub genes in different stages in patients with LUAD. In addition, we further analyzed the co-expression correlation between hub genes and the expression level of hub genes in different cancers using GEPIA2.

### Analysis of prognosis

The Kaplan-Meier Plotter is an online survival analysis database for 54,000 genes in 21 malignant tumors [16]. We used the Kaplan-Meier Plotter to determine the ability of the hub genes to predict OS of LUAD patients. In addition, we also analyzed the prognostic significance of the hub genes in different types of cancer using the GEPIA2 database.

### Analysis of immune infiltration, somatic mutation, and TMB score

We collected mRNA-seq data from 513 lung adenocarcinoma patients in the TCGA database. We then used the Timer algorithm to explore the infiltration of six immune cells, including B cell, macrophage, myeloid dendritic cell, neutrophil, CD4+ T cell CD4+, and T cell CD8+ in specific tumor tissues [14]. The multi-gene correlation map was displayed using the pheatmap and ggstatsplot packages in R software. To investigate the mutation frequency of hub genes in lung adenocarcinoma, we downloaded the hub gene mutation data from the TCGA database and visualized the somatic mutation landscape using the maftools package in R software. The ggstatsplot package in R software was used to explore the correlation between the TMB score and the hub genes.

## Results

### Identification of DEGs in LUAD

**Fig. 1:**
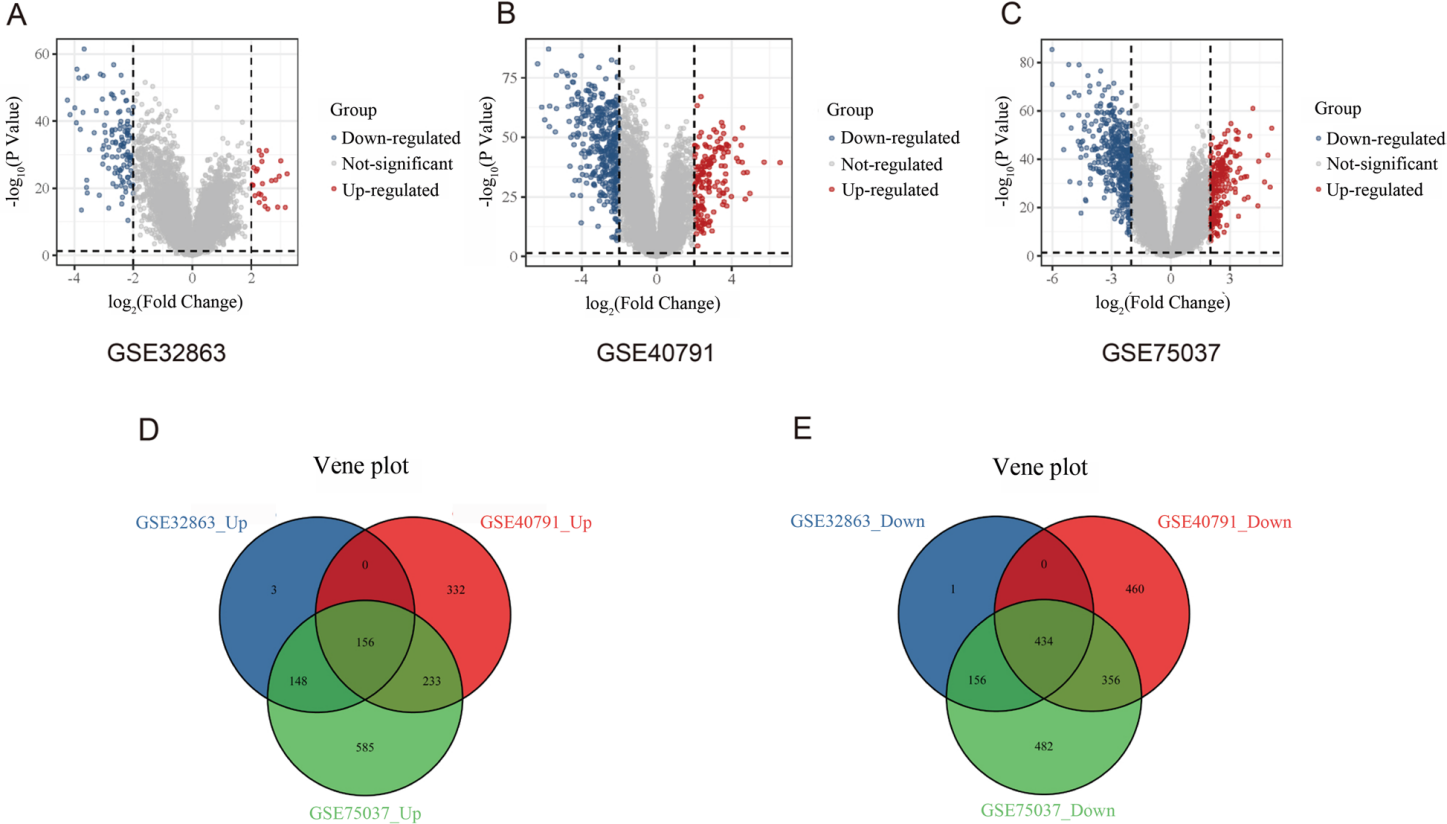
Screening of differentially expressed genes (DEGs). **A**–**C** Volcano maps showing the distribution of DEGs in three datasets. **D**, **E** Venn diagrams showing the intersection of common up-regulated and common down-regulated genes, respectively

We identified 307 up-regulated and 591 down-regulated DEGs in the GSE32863 dataset (Fig. 1A), 2298 DEGs including 721 up-regulated and 1250 down-regulated genes in the GSE40791 dataset (Fig. 1B), and 2550 DEGs including 1122 up-regulated and 1428 down-regulated genes in the GSE75037 dataset (Fig. 1C). Venn diagram analysis identified 156 common up-regulated genes and 434 down-regulated genes among the three GSE datasets (Fig. 1D, E).

### Gene ontology functional and pathway enrichment analysis of DEGs

**Fig. 2:**
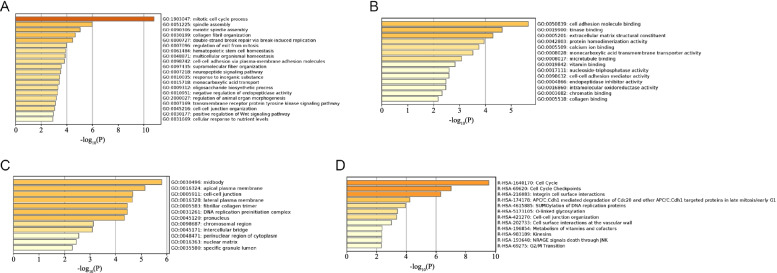
GO and pathway enrichment analysis of common up-regulated DEGs. **A**–**C** The enrichment analysis results of biological processes (BP), cellular components (CC), and molecular functions (MF). **D** The results of pathway enrichment analysis

In this study, Metascape was used to perform gene ontology functional annotation and pathway enrichment analysis of the common up-regulated genes from the three datasets. Gene ontology was explored according to the following three categories, namely biological processes (BP), cellular components (CC), and molecular functions (MF). BP terms were most significantly enriched in the mitotic cell cycle process, spindle assembly, meiotic spindle assembly, collagen fibril organization, and double-strand break repair via break-induced replication (Fig. 2A, Table 1). CC terms were enriched in midbody, apical plasma membrane, cell-cell junction, lateral plasma membrane, and fibrillar collagen trimer (Fig. 2B, Table 2). MF terms were most significantly enriched in cell adhesion molecule binding, kinase binding, extracellular matrix structural constituents, protein homodimerization activity, and calcium ion binding (Fig. 2C, Table 3). In addition, the results of pathway enrichment analysis revealed that all up-regulated DEGs were mainly enriched in cell cycle, cell cycle checkpoints, integrin cell surface interactions, APC/C:Cdh1-mediated degradation of Cdc20, and other APC/C:Cdh1-targeted proteins in late mitosis/early G1 and SUMOylation of DNA replication proteins (Fig. 2D, Table 4).

**Table 1:** The analysis of biological process enrichment

Term	Description	Count	LogP
GO:1903047	Mitotic cell cycle process	23	−10.799
GO:0051225	Spindle assembly	8	−5.971
GO:0090306	Meiotic spindle assembly	3	−5.039
GO:0030199	Collagen fibril organization	5	−4.645
GO:0000727	Double-strand break repair via break-induced replication	3	−4.451
GO:0007096	Regulation of exit from mitosis	3	−3.970
GO:0061484	Hematopoietic stem cell homeostasis	3	−3.893
GO:0048871	Multicellular organismal homeostasis	11	−3.860
GO:0098742	Cell-cell adhesion via plasma-membrane adhesion molecules	8	−3.807
GO:0097435	Supramolecular fiber organization	13	−3.559
GO:0007218	Neuropeptide signaling pathway	5	−3.500
GO:0010035	Response to inorganic substance	11	−3.428
GO:0015718	Monocarboxylic acid transport	5	−3.408
GO:0009312	Oligosaccharide biosynthetic process	3	−3.307
GO:0010951	Negative regulation of endopeptidase activity	7	−3.275
GO:2000027	Regulation of animal organ morphogenesis	5	−3.172
GO:0007169	Transmembrane receptor protein tyrosine kinase signaling pathway	11	−3.099
GO:0045216	Cell-cell junction organization	6	−3.037
GO:0030177	Positive regulation of Wnt signaling pathway	5	−2.951
GO:0031669	Cellular response to nutrient levels	6	−2.907

**Table 2:** The analysis of cellular component enrichment

Term	Description	Count	LogP
GO:0030496	Midbody	9	−5.802
GO:0016324	Apical plasma membrane	11	−5.142
GO:0005911	Cell-cell junction	12	−4.671
GO:0016328	Lateral plasma membrane	5	−4.645
GO:0005583	Fibrillar collagen trimer	3	−4.451
GO:0031261	DNA replication preinitiation complex	3	−4.451
GO:0045120	Pronucleus	3	−4.339
GO:0098687	Chromosomal region	8	−3.127
GO:0045171	Intercellular bridge	4	−3.091
GO:0048471	Perinuclear region of cytoplasm	11	−2.553
GO:0016363	Nuclear matrix	4	−2.465
GO:0035580	Specific granule lumen	3	−2.304

**Table 3:** The analysis of molecular function enrichment

Term	Description	Count	LogP
GO:0050839	Cell adhesion molecule binding	14	−5.655
GO:0019900	Kinase binding	15	−4.649
GO:0005201	Extracellular matrix structural constituent	7	−4.293
GO:0042803	Protein homodimerization activity	13	−3.950
GO:0005509	Calcium ion binding	13	−3.748
GO:0008028	Monocarboxylic acid transmembrane transporter activity	4	−3.516
GO:0008017	Microtubule binding	7	−3.080
GO:0019842	Vitamin binding	5	−2.831
GO:0017111	Nucleoside-triphosphatase activity	10	−2.596
GO:0098632	Cell-cell adhesion mediator activity	3	−2.594
GO:0004866	Endopeptidase inhibitor activity	5	−2.475
GO:0016860	Intramolecular oxidoreductase activity	3	−2.473
GO:0003682	Chromatin binding	9	−2.325
GO:0005518	Collagen binding	3	−2.175

**Table 4:** The analysis of significant pathway enrichment

Term	Description	Count	LogP
R-HSA-1640170	Cell cycle	21	−9.543
R-HSA-69620	Cell cycle checkpoints	12	−7.004
R-HSA-216083	Integrin cell surface interactions	7	−6.318
R-HSA-174178	APC/C:Cdh1 mediated degradation of Cdc20 and other APC/C:Cdh1 targeted proteins in late mitosis/early G1	5	−4.237
R-HSA-4615885	SUMOylation of DNA replication proteins	4	−3.946
R-HSA-5173105	O-linked glycosylation	5	−3.408
R-HSA-421270	Cell-cell junction organization	4	−3.352
R-HSA-202733	Cell surface interactions at the vascular wall	5	−2.993
R-HSA-196854	Metabolism of vitamins and cofactors	5	−2.375
R-HSA-193648	NRAGE signals death through JNK	3	−2.365
R-HSA-983189	Kinesins	3	−2.365
R-HSA-69275	G2/M Transition	5	−2.319

### PPI network construction and hub gene screening

**Fig. 3:**
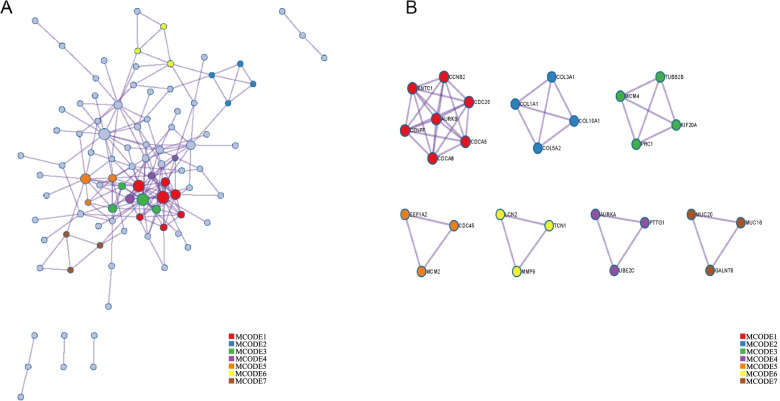
The protein-protein interaction (PPI) network constructed using common up-regulated DEGs. **A** The landscape of PPI network. **B** The significant subnetworks originating from the overall PPI network

The PPI network was constructed using Metascape and each code represented the specific common up-regulated DEG in the network (Fig. 3A). The molecular complex detection (MCODE) algorithm was applied to identify densely connected network components. In the whole screening process, seven clusters of MCODE with closely related functions were found and displayed in different colors, namely MCODE1, MCODE2, MCODE3, MCODE4, MCODE5, MCODE6, and MCODE7 (Fig. 3B). MCODE1 consisted of AURKB, CCNB2, KNTC1, CENPF, CDCA8, CDCA5, and CDC20; MCODE2 consisted of COL3A1, COL1A1, COL5A2, and COL10A1; MCODE3 consisted of TUBB2B, MCM4, PRC1, and KIF20A; MCODE4 consisted of EEF1A2, MCM2, and CDC45; MCODE5 consisted of LCN2, MMP9, and TCN1; MCODE6 consisted of AURKA, PTTG1, and UBE2C; MCODE7 consisted of MUC20, GALNT6, and MUC16, respectively. MCODE1 had the largest number of genes and further analysis was carried out to determine the clinical significance of each gene in LUAD.

### Expression analysis

**Fig. 4:**
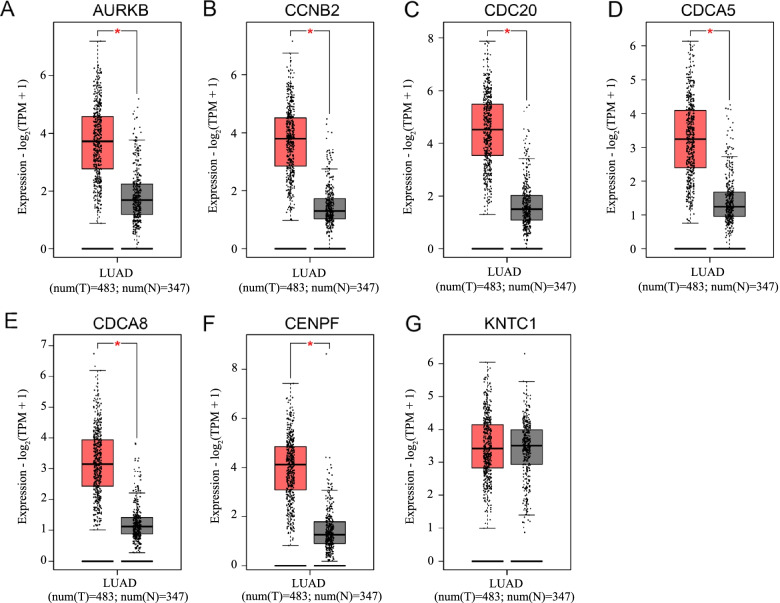
Comparison of the expression levels of hub genes between tumor tissues and normal tissues in LUAD patients using the GEPIA2 database. **A**–**G** The expression levels of AURKB, CCNB2, CDC20, CDCA5, CDCA8, CENPF, and KNTC1 in LUAD tissues are significantly higher in tumor tissues than in normal lung tissues

**Fig. 5:**
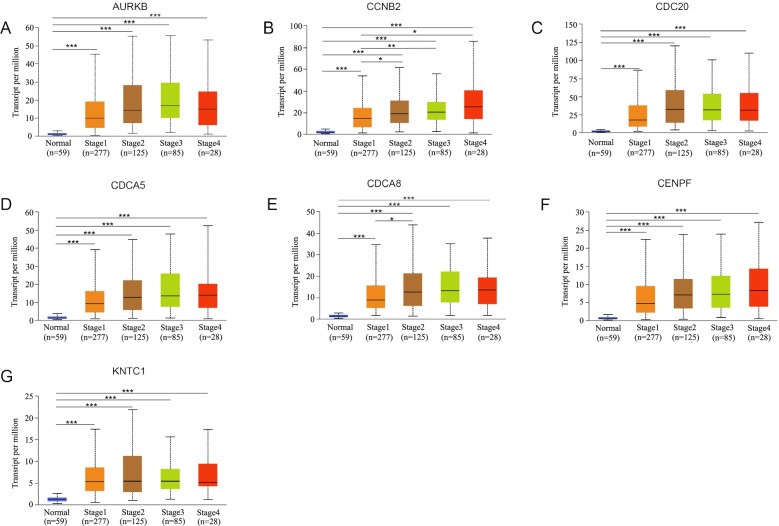
The analysis of expression levels of hub genes in different stages of LUAD patients using the UACALN database. **A**–**G** The expression levels of AURKB, CCNB2, CDC20, CDCA5, CDCA8, CENPF, and KNTC1 were significantly higher in tumor tissues than in normal tissues during the different stages of LUAD

Using the expression analysis function in the GEPIA2 database, we found that all the genes in MCODE1 apart from KNTC1 were significantly up-regulated in tumor tissues compared to normal samples (Fig. 4). Furthermore, we found that the expression levels of the seven genes were significantly higher in the tumor tissue than in normal tissues regardless of tumor stage using UALCAN (Fig. 5). The seven genes were also highly expressed in most cancers (Supplementary Fig. 1).

### Survival analysis

**Fig. 6:**
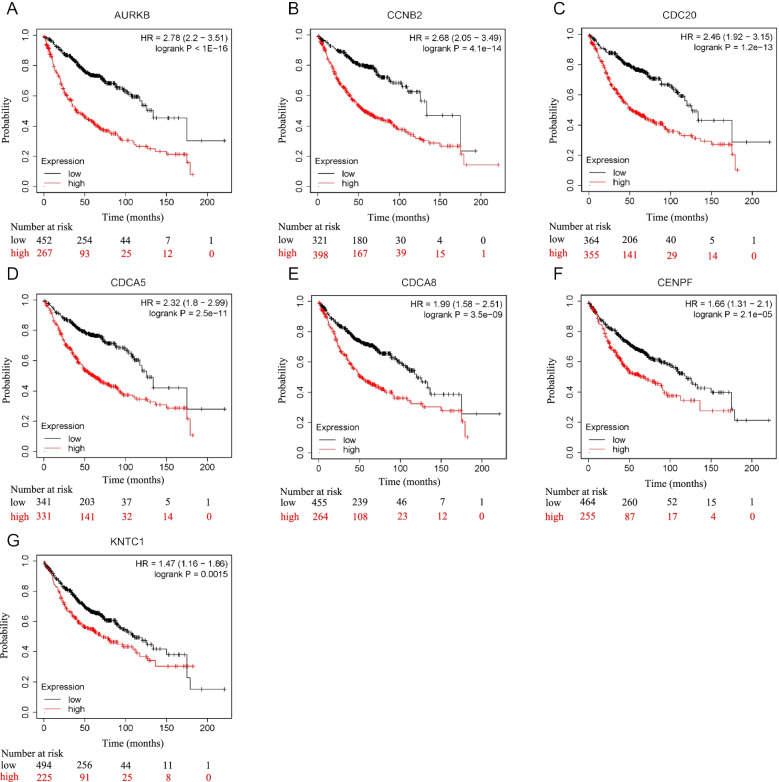
The survival analysis of hub genes based on the Kaplan-Meier database. **A**–**G** High expression levels of AURKB, CCNB2, CDC20, CDCA5, CDCA8, CENPF, and KNTC1 were associated with short OS in patients with LUAD

Kaplan-Meier analysis showed that high expression levels of the hub genes were associated with shorter overall survival of patients with LUAD (Fig. 6). The specific data is as follows: The *HR* of AURKA is 2.78, 95% *CI* is 2.2–3.51, and Logrank *P* is less than 1e−16; the *HR* of CCNB2 is 2.68, 95% *CI* is 2.05–3.49, and Logrank *P* = 4.1e−14; the *HR* of CDC20 is 2.46, 95% *CI* is 1.92–3.15, and Logrank *P* = 1.2e−13; the *HR* of CDCA5 is 2.32, 95% *CI* is 1.8–2.99, and Logrank *P* = 2.5e−11; the *HR* of CDCA8 is 1.99, 95% *CI* is 1.58–2.51, and Logrank *P* = 3.5e−09; the *HR* of CENPF is 1.66, 95% *CI* is 1.31–2.10, and Logrank *P* = 2.1e−05; the HR of KNTC1 is 1.47, 95% *CI* was 1.16–1.86, and Logrank *P* = 0.0015. High expression of the hub genes was also associated with poor prognosis in the majority of cancers, although the results in LUAD and lung squamous cell carcinoma were inconclusive (Supplementary Fig. 2).

### Analysis of immune infiltration, somatic mutation, and TMB score

**Fig. 7:**
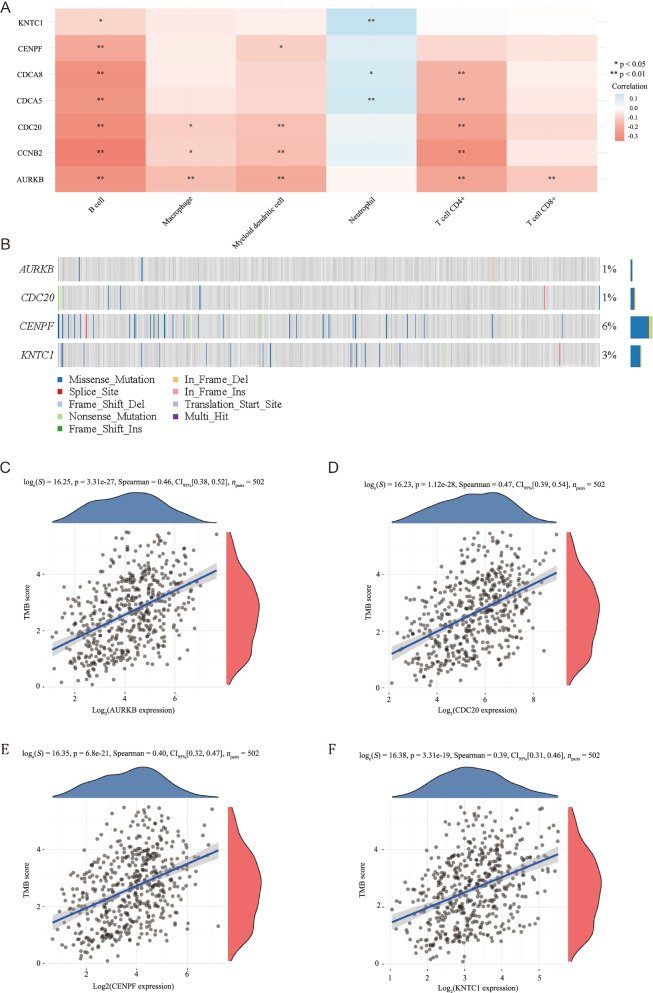
The correlation between the expression levels of hub genes and the infiltration of six immune cells and the TMB score in LUAD patients. **A** CDCA5 was positively correlated with B cell and CD4+ T cell; CDCA8 and KNTC1 were positively correlated with neutrophil; the rest of hub genes were negatively correlated with most immune cells. **B** Oncoplot displaying the somatic landscape of the LUAD cohort. The mutation frequencies of AURKB, CDC20, CENPF, and KNTC1 in LUAD patients were 1%, 1%, 6%, and 3%, respectively. In addition, missense mutation is the main mutation type. **C**–**F** Correlation analysis between hub gene expression and TMB score. AURKB, CDC20, CENPF, and KNTC1 were all positively correlated with the TMB score, and the Spearman correlation coefficients were 0.46, 0.47, 0.40, and 0.39, respectively

To investigate the potential function of the hub genes in LUAD patients, we used the TIMER algorithm to determine if the expression levels of the hub genes were associated with the infiltration levels of six types of immune cells in the tumor microenvironment of LUAD. AURKB was negatively correlated with B cell, macrophage, myeloid dendritic cell, CD4+ T cell, and CD8+ T cell infiltration; CCNB2 was negatively correlated with B cell, macrophage, myeloid dendritic cell, and CD4+T cell infiltration; CDC20 was negatively correlated with B cell, macrophage, myeloid dendritic cell, and CD4+ T cell infiltration; CDCA5 was negatively correlated with neutrophil, but positively correlated with B cell and CD4+T cell infiltration; CDCA8 was negatively correlated with B cell and CD4+ T cell, but positively correlated with neutrophil infiltration; CENPF were negatively correlated with B cell and myeloid dendritic cell infiltration; KNTC1 was negatively correlated with B cell but positively correlated with neutrophil infiltration (Fig. 7A, Table 5).

**Table 5:** The analysis of the correlation between immune cell infiltration and hub gene expression level in LUAD patients

Symbol	Variable	Correlation	*P* value
AURKB	B cell	−0.269	<0.001
AURKB	Macrophage	−0.147	<0.001
AURKB	Myeloid dendritic cell	−0.200	<0.001
AURKB	T cell CD4+	−0.281	<0.001
AURKB	T cell CD8+	−0.045	<0.05
CCNB2	B cell	−0.343	<0.001
CCNB2	Macrophage	−0.095	<0.05
CCNB2	Myeloid dendritic cell	−0.143	<0.01
CCNB2	T cell CD4+	−0.281	<0.001
CDC20	B cell	−0.199	<0.001
CDC20	Macrophage	−0.116	<0.05
CDC20	Myeloid dendritic cell	−0.107	<0.01
CDC20	T cell CD4+	−0.152	<0.001
CDCA5	B cell	0.264	<0.001
CDCA5	Neutrophil	−0.115	<0.01
CDCA5	T cell CD4+	0.177	<0.001
CDCA8	B cell	−0.281	<0.001
CDCA8	Neutrophil	0.100	<0.05
CDCA8	T cell CD4+	−0.175	<0.001
CENPF	B cell	−0.207	<0.001
CENPF	Myeloid dendritic cell	−0.104	<0.05
KNTC1	B cell	−0.088	<0.05
KNTC1	Neutrophil	0.146	<0.001

Somatic mutation analysis showed that AURKB, CDC20, CENPF, and KNTC1 had different types of mutations in patients with lung adenocarcinoma, and the main type was missense mutation (Fig. 7B). In addition, an increase in the expression of the four genes was associated with an increase in the TMB score (Fig. 7C–F). High TMB scores are associated with good response to immune therapy [17].

## Discussion

LUAD is one of the most important subtypes of non-small cell lung cancer, with high incidence and mortality [18]. Currently, there are no effective biomarkers for the accurate diagnosis of LUAD patients [19]. Therefore, we used bioinformatics tools to screen candidate genes for the diagnosis and prognosis of LUAD from the GEO database. In addition, we explored the correlation among the immune cell infiltration and TMB score and the expression levels of these genes in the LUAD tissues.

First, we identified 156 common up-regulated genes and 434 common down-regulated genes among the three GSE datasets obtained from the GEO database. The 156 common up-regulated genes were then submitted to Metascape for GO and pathway enrichment analysis. Biological processes (BP), molecular functions (MF), and cellular components (CC) were included in the GO analysis. Subsequently, the PPI network was constructed based on common up-regulated genes using Metascape and the MCODE plug-in was used to screen out seven important subnetworks in the PPI network, namely MCODE 1, MCODE 2, MCODE 3, MCODE 4, MCODE 5, MCODE 6, and MCODE 7 [20]. MCODE 1 contained the largest number of key genes, including AURKB, CDC20, CDCA5, CDCA8, CENPF, KNTC1, and CCNB2. Expression analysis using GEPIA2 and UALCAN databases showed that all the hub genes in MCODE 1 except KNTC1 were highly expressed in LUAD tissues compared to normal tissues. In addition, survival analysis using the Kaplan-Meier Plotter database showed that high expression of these key genes was associated with poor prognosis in patients with LUAD. Furthermore, we explored the correlation of these candidate genes with the infiltration of six immune cells in patients with LUAD to determine the potential response of the tumors to immunotherapy. It is worth noting that through somatic mutation analysis, we found that AURKB, CDC20, CENPF, and KNTC1 had different frequencies of mutations in patients with lung adenocarcinoma, and mainly missense mutation type. Furthermore, we analyzed the correlation between the expression of four genes and the TMB score and found that with the increase of the expression of these genes in patients with lung adenocarcinoma, the TMB score also increased. It is well known that a high TMB score indicates that cancer patients have better immunotherapy effects. Therefore, if we can improve the proportion of immune cell infiltration in lung adenocarcinoma tissues in future clinical studies, it may be able to effectively improve the efficacy of immunotherapy and prolong the survival period of patients with lung adenocarcinoma.

AURKB (aurora kinase B) is a protein-coding gene that acts as a key regulator of mitosis [21]. Many studies have confirmed that AURKB is a crucial carcinogenic factor in different kinds of carcinoma. For example, AURKB was found to be expressed at higher levels in renal cell carcinoma tissues than in normal kidney tissues, suggesting that it may regulate renal cell carcinoma progression by modulating the intestinal immune network for IgA production and signaling pathways involving cytokine-cytokine receptor interactions [22]. Furthermore, AURKB was overexpressed in gastric cancer and was strongly linked to clinicopathological features of the disease. Silencing of AURKB may decrease the invasive and migratory capacities of gastric cancer cells by disrupting the VEGFA/Akt/mTOR and Wnt/-catenin/Myc pathways [23]. Additionally, AURKB activation was associated with acquired resistance to EGFR TKIs, suggesting that AURKB should be targeted in NSCLC patients scheduled for anti-EGFR treatment but who lack resistance mutations [24].

CDC20 (cell division cycle 20) appears to act as a regulatory protein interacting with several other proteins at multiple points in the cell cycle [25]. Min Shi et al. found that CDC20 played a crucial role in the development of hepatocellular carcinoma by regulating the PHD3 protein [26]. Besides, Yang Gao et al. found that targeting CDC20 sensitized colorectal cancer cells to radiotherapy through mitochondrial-dependent apoptotic signaling [27]. Qin Zhang et al. found that CDC20 combined with CD44 or β-catenin could serve as an important indicator for the prognosis of patients with prostate cancer [28]. Furthermore, Huan Deng et al. found that CDC20 was up-regulated in LUSC at the mRNA and protein levels [29]. However, the role and mechanisms of action of CDC20 in LUAD remain unclear.

CDCA5 (cell division cycle associated 5) is another protein-coding gene involved in DNA repair [30]. CDCA5 promotes the progression of bladder cancer by dysregulating mitochondria-mediated apoptosis, cell cycle regulation, and activation of the PI3k/AKT/mTOR pathway [31]. Additionally, CDCA5 aids in the development of esophageal squamous cell carcinoma and may be an important target for esophageal squamous cell carcinoma immunotherapy [32]. Moreover, CDCA5 phosphorylation and activation by mitogen-activated protein kinase are critical for human lung cancer [33].

CDCA8 (cell division cycle associated 8) is a component of the chromosomal passenger complex, which is required for mitosis and cell division [34]. Increased CDCA8 expression in ovarian tissues probably plays a critical role in the development of ovarian cancer through the PLK1 pathway [35]. Besides, CDCA8 is involved in the construction of meiotic spindles and chromosomal segregation during human oocyte meiosis [36]. CDCA8 overexpression has been shown to accelerate the development of cutaneous melanoma and is associated with poor prognosis [37]. Additionally, aurora kinase B-mediated phosphorylation and activation of CDCA8 plays a major role in human lung cancer [38]. Moreover, miR-133b suppressed cell proliferation, motility, and invasion in lung adenocarcinoma by targeting CDCA8 [39].

CENPF is a gene that encodes a protein that is involved in the centromere-kinetochore complex association [40]. Overexpression of CENPF in breast cancer was associated with poor prognosis and tumor bone metastases by controlling parathyroid hormone-related peptide (PTHrP) production via activating PI3K-AKT-mTORC1 [41]. Besides, the HnRNPR-CCNB1/CENPF axis was involved in the proliferation and metastasis of gastric cancer [42]. Additionally, silencing CENPF substantially reduced LUAD cell tumor development in an experimental xenograft lung cancer model using naked mice armpits of the right forelimb. However, there are no sufficient studies on the mechanism of CENPF in LUAD [43].

KNTC1 encodes a protein participating in the processes that guarantee correct chromosomal segregation during cell division [44]. A recent study indicated that silencing KNTC1 with shRNA inhibited cell viability and caused apoptosis in esophageal squamous cell carcinoma [45]. Moreover, relevant bioinformatics publications demonstrated that KNTC1 was associated with a poor outcome in patients with hepatocellular carcinoma and cervical cancer [46, 47]. However, there has been no research on the mechanism of action of KNTC1 in patients with LUAD.

CCNB2 (cyclin B2) is a member of the B-type cyclins family that can interact with p34cdc2, and is a critical component of the cell cycle regulation [48]. CCNB2 has been shown to promote the proliferation of triple-negative breast cancer cells in vitro and in vivo [49]. As validated by a comprehensive bioinformatics study, CCNB2 has been identified as a promising therapeutic target for ovarian cancer [50]. Additionally, miR-335-5p disrupts the cell cycle and increases lung adenocarcinoma metastasis by targeting CCNB2 [51]. Moreover, CCNB2 had been identified as a marker of responsiveness to immune checkpoint inhibitors (ICI) in NSCLC and overexpression of CCNB2 is a poor prognostic indicator in Chinese patients with NSCLC [52, 53].

In recent years, an increasing number of studies have revealed that diverse immune components in the tumor microenvironment play a significant role in the molecular process of various tumors and development. A study on the advanced lung cancer inflammation index, for example, discovered that it can predict shorter overall survival not only in patients with lung adenocarcinoma, but also in patients with other tumors at a low level of expression [54]. TMED2, MOESIN, DPYSL2, and LncRNA MEG3 have been discovered to enhance the occurrence and development of patients with lung adenocarcinoma via several immune-related regulatory mechanisms [55–58]. Furthermore, the hunt for efficient immune checkpoint inhibitors and indicators to predict the efficacy of immunotherapy is critical. It is well known that pembrolizumab and chemotherapy are currently the first-line treatments for small cell lung cancer, and their therapeutic effectiveness has been widely accepted [59]. In the studies related to lung adenocarcinoma patients, it was found that individuals with smoking history could benefit more from the treatment of immune checkpoint inhibitors and that the combination of immune checkpoint inhibitors nivolumab and ipilimumab was much more effective than monotherapy [60, 61]. However, there is currently a scarcity of effective indicators of tumor immunotherapy. We expect that further indicators similar to PD-L1 will emerge in the future to predict the efficacy of NSCLC immunotherapy [62]. Therefore, we expect that the results of this study will provide reference value to the immunotherapy of lung adenocarcinoma patients from different molecular subtypes. The findings of our research were acquired through data mining of an online database using bioinformatics techniques. The limitations of this study include the absence of specific in vitro or in vivo experiments to validate the significance of the selected hub genes in LUAD patients. Additionally, the results of our study may include partial bias owing to the issue of data quantity and quality. As a result, there is a need for further studies to validate our findings and identify the role of these genes in lung adenocarcinoma.

## Conclusion

In conclusion, the high expression of the candidate genes screened in this study is associated with poor prognosis in LUAD patients. High expression of the candidate genes combined with the TMB score indicates a better response to immunotherapy in patients with lung adenocarcinoma. However, there is a need for more experiments to validate the significance and mechanism of action of these genes in LUAD patients.

## Supplementary Information

**Additional file 1: Supplementary Fig. 1.** The expression landscape of hub genes in different cancers using GEPIA2 database. The graph demonstrated that the hub genes were highly expressed in the majority of cancers.

**Additional file 2: Supplementary Fig. 2.** The prognostic values of the hub genes in different cancers. The higher the red intensity of the square color, the higher the gene expression level, indicating a worse prognosis for patients.

**Additional file 3: Supplementary Table 1.** Up and down regulation of differentially expressed genes (DEGs) in three GSE datasets.

## Data Availability

The datasets used and/or analyzed during the current study are available from the corresponding author on reasonable request.

## Abbreviations

LUAD: Lung adenocarcinoma
DEGs: Differentially expressed genes
GO: Gene ontology
PPI: Protein-protein interaction
MCODE: Molecular complex detection
MF: Molecular function
BP: Biological process
CC: Cellular component
GEO: Gene Expression Omnibus
OS: Overall survival
AURKB: Aurora kinase B
CDC20: Cell division cycle 20
CDCA5: Cell division cycle associated 5
CDCA8: Cell division cycle associated 8
CCNB2: Cyclin B2
NSCLC: Non-small cell lung cancer
ICI: Immune checkpoint inhibitor
