# Supplementary figures and images for: An integrated analysis of prognostic and immune infiltrates for hub genes as potential survival indicators in patients with lung adenocarcinoma
Source: World J Surg Oncol. 2022 Mar 30;20:99. doi: 10.1186/s12957-022-02543-z (PMC8966338; doi:10.1186/s12957-022-02543-z)

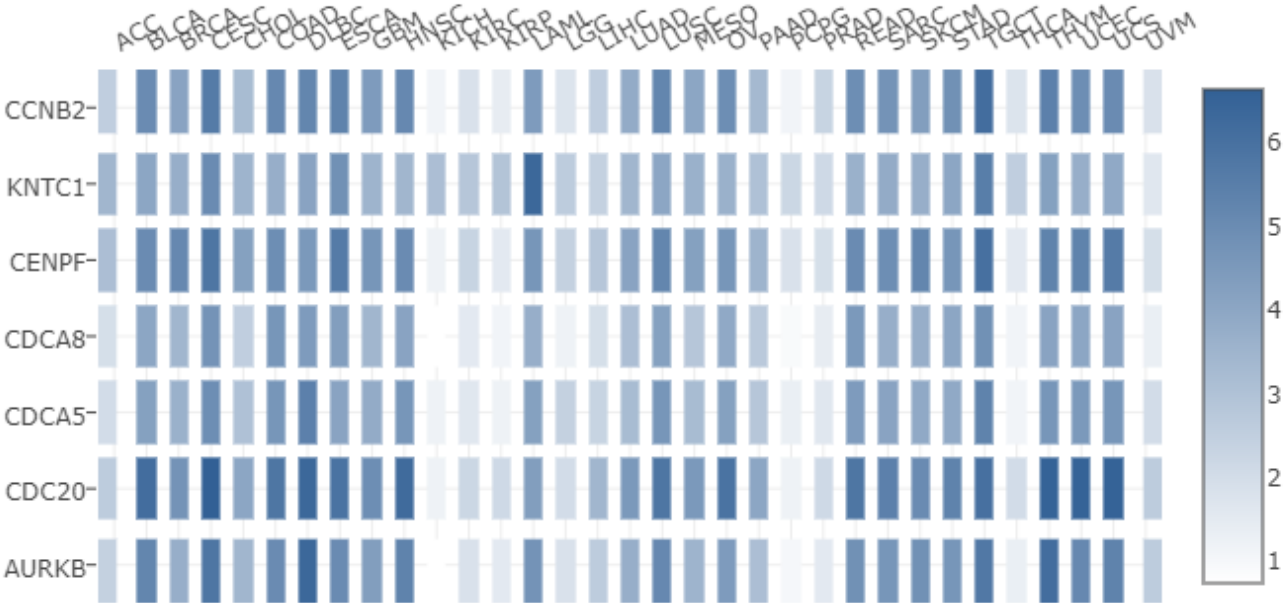

Supplement: Supplementary file 1 — Additional file 1: Supplementary Fig. 1. The expression landscape of hub genes in different cancers using GEPIA2 database. The graph demonstrated that the hub genes were highly expressed in the majority of cancers. [file 12957_2022_2543_MOESM1_ESM.pdf]

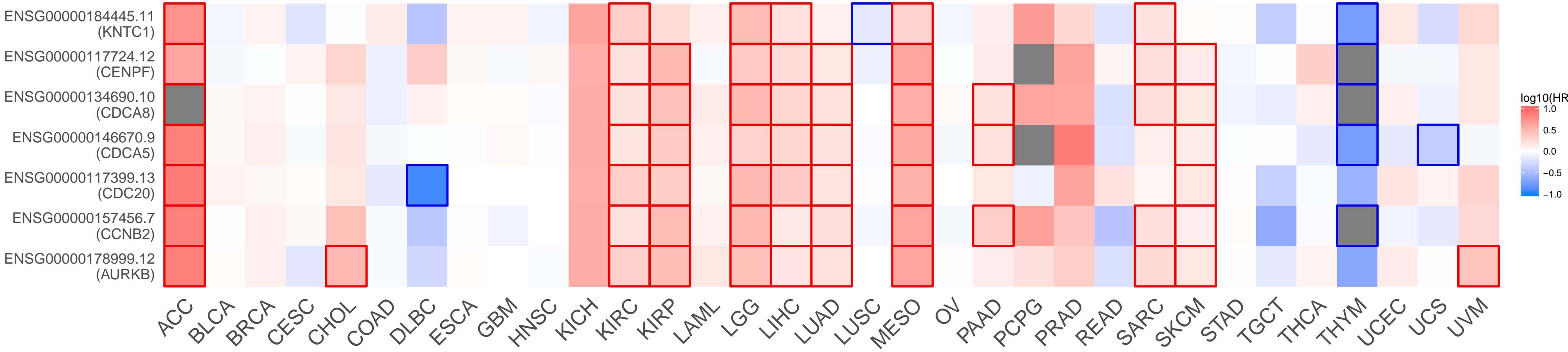

Supplement: Supplementary file 2 — Additional file 2: Supplementary Fig. 2. The prognostic values of the hub genes in different cancers. The higher the red intensity of the square color, the higher the gene expression level, indicating a worse prognosis for patients. [file 12957_2022_2543_MOESM2_ESM.pdf]
