# Supplementary material for: An integrated analysis of prognostic and immune infiltrates for hub genes as potential survival indicators in patients with lung adenocarcinoma
Source: World J Surg Oncol. 2022 Mar 30;20:99. doi: 10.1186/s12957-022-02543-z (PMC8966338; doi:10.1186/s12957-022-02543-z)
Supplement: Supplementary file 3 — Additional file 3: Supplementary Table 1. Up and down regulation of differentially expressed genes (DEGs) in three GSE datasets. [file 12957_2022_2543_MOESM3_ESM.docx]

**Supplementary Table1** Up and down regulation of differentially expressed genes (DEGs) in three GSE datasets.

| Up-regulated | Down-regulated |
| --- | --- |
| BAIAP2L1, ETV4, TOP2A, GPT2, SRPK1, SLC39A11, CNOT11, SLC2A1, KRT8, OCIAD2, HOOK1, IGSF9, INAVA, TNFRSF21, SLC52A2, STIL, TK1, CDC20, ERO1A, SFN, PAFAH1B3, AURKA, GCNT3, CDCA5, PDIA4, SPP1, MARCKSL1, PRC1, CRABP2, ALDH18A1, SOX4, BZW2, ABCC3, COL1A1, TMEM45B, UBE2T, TMEM184A, XPR1, SLC50A1, ARHGEF16, ELMO3, MCM4, CCNB2, SLC16A3, STK39, TLCD1, GFPT1, P3H4, CDH3, GSDMB, UBE2C, CDH1, KIF20A, CDCA8, FAM83A, ASPM, RAB25, CENPF, FUT3, ANKRD22, SHMT2, MELK, MCM2, FUT2, METTL7B, SAPCD2, AURKB, PLEK2, GLB1L2, CDC45, THBS2, SMPDL3B, CAPN12, CEACAM1, MMP9, KIFC1, KNTC1, MUC20, SLC35F2, ARHGEF19, RMI2, ECT2, HMGB3, PTGES, COL10A1, TMPRSS4, NPM3, SIX4, COMP, TPX2, CTHRC1, GALNT6, RGS17, LAPTM4B, WFDC3, CST1, SLC7A5, EEF1A2, SPINK1, CLDN3, C4ORF48, ENC1, CEACAM5, PTTG1, MX2, GGCT, COL5A2, TYMS, RASEF, XKRX, KCNK1, NRIP3, GPR37, ZNF750, REEP6, COL3A1, PCSK1N, GJB2, NMU, SCG5, DNTTIP1, AGR2, CHRNA5, DNAJC12, SULF1, VSTM2L, FOXA3, SLC16A9, RHPN2, IGF2BP3, TRIP13, KISS1R, HABP2, THY1, CFB, ST6GALNAC1, AOC1, LCN2, IGFBP3, PGGHG, DDIT4L, PCP4, AHNAK2, TUBB2B, MUC16, KRT15, CXCL14, GPX2, CRLF1, GFRA3, TM4SF4, S100P, TCN1, AKR1B10, CLDN2, TFF1 | FABP4, ADH1B, FMO2, TCF21, AGER, CAV1, FAM107A, CLEC1A, RHOJ, STX11, JAM2, RGCC, LDB2, TEK, ABCA8, PLAC9, MCEMP1, LYVE1, PECAM1, CCM2L, GIMAP6, S1PR1, CYYR1, CA4, MMRN1, GHR, MFAP4, FHL1, KANK3, ADH1A, HIGD1B, SPOCK2, CD36, LIMS2, F10, HBB, GSTM5, TM6SF1, PTPRB, SH3GL3, COX7A1, CDH5, HSPB2, OLFML1, ADAMTS8, CRYAB, CCL14, HSPA12B, MFNG, CD93, GLIPR2, LAMP3, COL6A6, HBA1, SPARCL1, CPA3, DPEP2, CAV2, CDO1, TGFBR3, MAMDC2, SRPX, HEG1, CFD, INMT, ABI3BP, C1ORF162, CNRIP1, CAVIN2, EPAS1, VWF, SCARA5, EMCN, ACVRL1, PGM5, VSIG4, CPED1, CD300LG, GDF10, C14ORF132, MS4A7, GRK5, ITM2A, PLPP3, SLC31A2, DNASE1L3, VSIR, CRTAC1, AQP4, PTPRM, ALKAL2, MS4A2, FXYD1, TNNC1, PPP1R14A, RASL12, CD52, FGD5, FXYD6, CLDN5, PTGER4, GPC3, CLEC14A, ANOS1, PCOLCE2, ADIRF, ADRB2, FCN1, CXCL12, PEBP4, LGI3, SLC19A3, C5AR1, CAT, FBLN5, ACADL, TMEM100, ANGPT1, GKN2, GMFG, RHEX, DCN, GPER1, GIMAP4, TMEM88, KLF4, FAM189A2, GPM6B, FGR, SEMA6A, FZD4, SNCA, ARHGEF6, CALCRL, SELENOP, BCHE, PID1, FOXF1, RASIP1, EMP3, FAM162B, TACC1, MT1M, HOXA5, IL33, AOC3, SOX17, C1ORF21, TIMP3, GIMAP8, INKA1, RAMP3, ACKR1, CLDN18, HSPB6, LPL, MARCO, SOSTDC1, CGNL1, AQP9, HBEGF, FERMT2, STXBP6, EFEMP1, SLIT3, KL, TMEM74B, PPP1R15A, MSRB3, COL13A1, TGFBR2, RRAS, HSPB8, CCN5, GPX3, ECRG4, CES1, CCL23, TUBB6, FABP5, TOX2, RTKN2, DUOX1, CD33, ECM2, RAMP2, LTBP4, ZNF106, IL17D, LMCD1, VGLL3, ARHGAP44, FCN3, ID3, JAML, TMEM47, WASF3, ALOX5AP, LMO2, GJA4, SPTBN1, FBLN1, SASH1, LRRC32, LHFPL6, ITPRIP, TMEM204, TCEAL2, NDRG2, PDK4, GNG11, CACNA2D2, RIPOR1, S100A8, CAVIN1, DPT, OLR1, MYH10, COLEC12, C7, PLSCR4, SLCO2B1, NTNG1, CDH13, ECSCR, PTGDS, ADAMTS1, CD69, RILPL2, SCN4B, ESAM, C1QA, SOCS2, ADCY4, RAB8B, VEGFD, CBX7, IL6, PTH1R, SOX7, SLC7A7, EGR2, CPB2, KLF2, CD300LF, FCER1A, FCER1G, OSR1, TYROBP, RNF144B, AOX1, SLC46A2, PRG4, CTNNAL1, UBE2E2, SMAD6, TSPAN7, SLIT2, GLDN, ZFP36, RARRES2, CYBRD1, FMO3, FGFBP2, DUOXA1, ENPP2, ANKRD29, LST1, GATA2, DUSP1, COX4I2, CLIC5, RETN, DPYSL2, MNDA, NOSTRIN, HCK, KLF6, S100A4, ICAM2, OSCAR, NPNT, LIMCH1, WFDC1, SH2B3, CYP4B1, ANXA3, STARD8, VIPR1, DES, GSTM3, ALDH1A2, HSD17B6, PROS1, TPSAB1, MYL9, BTG2, PLA2G1B, SHANK3, ABCA3, ADTRP, KLF9, SFTPC, PRICKLE2, WIF1, TMOD1, ZBED2, S100A3, SLC6A4, TRPV2, DENND2A, CNN1, GAS1, SPON1, LRRC36, GADD45B, KCNJ8, ETS1, NDN, SOX18, NOTCH4, SGCE, PGC, TPPP3, CA2, SLC15A3, ACTG2, EFEMP2, GABARAPL1, FAM167A, ADGRL2, CYP27A1, CCL21, MAL, FAM43A, FOSB, WFS1, IL7R, SLC39A8, C1QB, CSRNP1, MYH11, IFT57, SLPI, PDLIM3, CTSG, CD83, FOLR3, ALDH1A1, LYZ, PTGIS, ENG, AK1, C8B, INAFM1, RERG, ALDH2, EMP1, LAMC3, RGS2, SBSPON, CLIC3, ID1, CPVL, SLCO2A1, CCN2, EPB41L3, OLFML2A, PAPSS2, MS4A15, C1ORF198, SGK1, SFTPD, NKD2, C1ORF115, MTURN, GALNT18, PRNP, GBP4, CSF3R, CAVIN3, NKG7, PPP1R3C, SUSD2, PLAC8, GZMH, RGS5, P3H2, LDLR, SCGB1A1, SLC2A3, AQP1, AADAC, PLLP, ALPL, PHACTR2, DEFA1, EGR1, NDNF, FOS, IGFBP6, FAM216B, TBX2, NUPR1, TEKT1, C20ORF85, C9ORF24, FBP1, C2, JUN, ZNF385B, SCGB3A2, SFTPB, ZBTB16, NEDD4L, VSIG2, MAOA, JUNB, ACP5, C6, DYNLRB2, GPRC5A, ANG, CCN1, NINJ2, C4BPA, TMSB15A, GJA1, EDN1, ATOH8, MS4A8, SCGB3A1, CAMK2N1, HPGD |
